# Metabolite profiling of CKD progression in the chronic renal insufficiency cohort study

**DOI:** 10.1172/jci.insight.161696

**Published:** 2022-10-24

**Authors:** Donghai Wen, Zihe Zheng, Aditya Surapaneni, Bing Yu, Linda Zhou, Wen Zhou, Dawei Xie, Haochang Shou, Julian Avila-Pacheco, Sahir Kalim, Jiang He, Chi-Yuan Hsu, Afshin Parsa, Panduranga Rao, James Sondheimer, Raymond Townsend, Sushrut S. Waikar, Casey M. Rebholz, Michelle R. Denburg, Paul L. Kimmel, Ramachandran S. Vasan, Clary B. Clish, Josef Coresh, Harold I. Feldman, Morgan E. Grams, Eugene P. Rhee, the CKD Biomarkers Consortium and CRIC Study Investigators

**Affiliations:** 1 Nephrology Division and; 2 Endocrine Unit, Massachusetts General Hospital, Boston, Massachusetts, USA.; 3 Department of Biostatistics, Epidemiology, and Informatics, Perelman School of Medicine, University of Pennsylvania, Philadelphia, Pennsylvania, USA.; 4 Department of Epidemiology, Johns Hopkins University Bloomberg School of Public Health, Baltimore, Maryland, USA.; 5 Welch Center for Prevention, Epidemiology, and Clinical Research, Johns Hopkins University, Baltimore, Maryland, USA.; 6 Department of Epidemiology, Human Genetics & Environmental Sciences, University of Texas Health Sciences Center at Houston School of Public Health, Houston, Texas, USA.; 7 Broad Institute of MIT and Harvard, Cambridge, Massachusetts, USA.; 8 Department of Epidemiology, Tulane University School of Public Health and Tropical Medicine, New Orleans, Louisiana, USA.; 9 Division of Nephrology, University of California San Francisco School of Medicine, San Francisco, California, USA.; 10 Division of Research, Kaiser Permanente Northern California, Oakland, California, USA.; 11 Division of Kidney, Urologic, and Hematologic Diseases, National Institute of Diabetes and Digestive and Kidney Diseases (NIDDK), Bethesda, Maryland, USA.; 12 Division of Nephrology, University of Michigan Medical School, Ann Arbor, Michigan, USA.; 13 Division of Nephrology and Hypertension, Wayne State University School of Medicine, Detroit, Michigan, USA.; 14 Renal-Electrolyte and Hypertension Division, Perelman School of Medicine, University of Pennsylvania, Philadelphia, Pennsylvania, USA.; 15 Section of Nephrology, Boston University School of Medicine, Boston Medical Center, Boston, Massachusetts, USA.; 16 Division of Pediatric Nephrology, Children’s Hospital of Philadelphia, and; 17 Center for Clinical Epidemiology and Biostatistics, Perelman School of Medicine, University of Pennsylvania, Philadelphia, Pennsylvania, USA.; 18 Department of Epidemiology, Boston University School of Public Health, Boston, Massachusetts, USA.; 19 Sections of Preventive Medicine and Epidemiology and Cardiology, Department of Medicine, Boston University School of Medicine, Boston, Massachusetts, USA.; 20 Department of Medicine, New York University, New York, New York, USA.; 21 The CKD Biomarkers Consortium and the CRIC Study Investigators are detailed in Supplemental Acknowledgments.

**Keywords:** Nephrology, Chronic kidney disease

## Abstract

**BACKGROUND:**

Metabolomic profiling in individuals with chronic kidney disease (CKD) has the potential to identify novel biomarkers and provide insight into disease pathogenesis.

**METHODS:**

We examined the association between blood metabolites and CKD progression, defined as the subsequent development of end-stage renal disease (ESRD) or estimated glomerular filtrate rate (eGFR) halving, in 1,773 participants of the Chronic Renal Insufficiency Cohort (CRIC) study, 962 participants of the African-American Study of Kidney Disease and Hypertension (AASK), and 5,305 participants of the Atherosclerosis Risk in Communities (ARIC) study.

**RESULTS:**

In CRIC, more than half of the measured metabolites were associated with CKD progression in minimally adjusted Cox proportional hazards models, but the number and strength of associations were markedly attenuated by serial adjustment for covariates, particularly eGFR. Ten metabolites were significantly associated with CKD progression in fully adjusted models in CRIC; 3 of these metabolites were also significant in fully adjusted models in AASK and ARIC, highlighting potential markers of glomerular filtration (pseudouridine), histamine metabolism (methylimidazoleacetate), and azotemia (homocitrulline). Our findings also highlight N-acetylserine as a potential marker of kidney tubular function, with significant associations with CKD progression observed in CRIC and ARIC.

**CONCLUSION:**

Our findings demonstrate the application of metabolomics to identify potential biomarkers and causal pathways in CKD progression.

**FUNDING:**

This study was supported by the NIH (U01 DK106981, U01 DK106982, U01 DK085689, R01 DK108803, and R01 DK124399).

## Graphical abstract

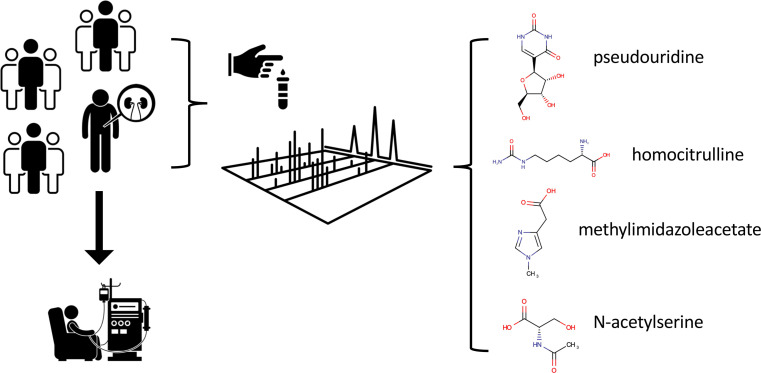

## Introduction

Metabolomics refers to the systematic analysis of small molecules (typically < 1,500 daltons) in a biologic specimen (1). Because metabolites (e.g., sugars, amino acids, organic acids, nucleotides, lipids) are downstream of transcriptional and translational processes, they represent the summative effects of gene and protein expression and interaction with the environment. Characterized by the progressive retention of small molecules, chronic kidney disease (CKD) is particularly well suited for metabolomic interrogation. Given estimates that > 10% of the US population has CKD (2), the identification of noninvasive metabolite markers of disease progression — ideally linked to disease pathogenesis — represents an important opportunity to improve estimation of CKD prognosis and point to potential pathways that may be targeted for novel drug therapy.

The interaction between the kidney and circulating metabolites is complex. Metabolites like urea and creatinine primarily undergo filtration at the glomerulus, but they also undergo subsequent tubular absorption and secretion, respectively. Metabolites can also be synthesized by the tubular epithelium, as with 1,25-dihyroxyvitamin D. Classic studies of plasma sampling from the renal artery and vein have shown that the kidneys are also responsible for the net production of several amino acids (3, 4). Finally, circulating metabolites can drive biological processes in the kidney, as bioenergetic fuels, ligands for specific receptors, substrates for posttranslational modifications, or chemoattractants that trigger inflammation (5). Thus, metabolite alterations in CKD can signify impair filtration, absorption, secretion, or metabolism, as well as act as functional mediators of disease.

In the current study, we applied liquid chromatography–mass spectrometry (LC-MS) based profiling to identify metabolite markers of CKD progression in the Chronic Renal Insufficiency Cohort (CRIC) study, with replication in the African-American Study of Kidney Disease and Hypertensin (AASK) and the Atherosclerosis Risk in Communities (ARIC) study (6–9). These analyses spanned over 8,000 individuals with a broad range of baseline kidney function, age, race, sex, geography, and comorbidities. In our analyses, statistical models were serially adjusted to assess how covariates impact the association of metabolites with CKD progression, highlighting, in particular, the impact of confounding by glomerular filtration rate in CKD biomarker discovery. Furthermore, cross-sectional analyses were performed to examine the correlation of metabolite predictors of CKD progression with baseline measures of kidney function. Finally, exploratory animal studies informed by the metabolomics results in patient cohorts were conducted to demonstrate a potential causal role for a select metabolic pathway in kidney disease pathogenesis.

## Results

### Description of study cohorts.

**Figure 1:**
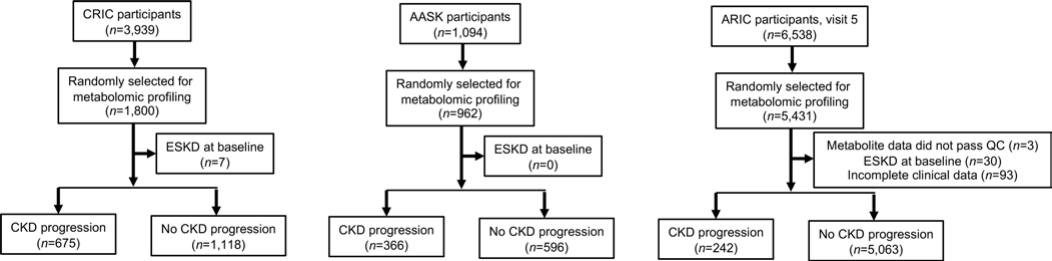
CKD progression across the study cohorts. Flow diagrams depicting the total number of study participants, the number of study participants selected for metabolomic profiling, exclusions from data analysis, and the number of study participants who did or did not have CKD progression in the CRIC Study (left), AASK (middle), and the ARIC Study (right). ESKD, end-stage kidney disease.

Characteristics of the study sample are shown in Table 1, with 1,773 CRIC Study participants, 962 AASK study participants, and 5,305 ARIC study participants. Mean age (58.8 [CRIC] and 54.6 years [AASK]) and estimated glomerular filtration rate (eGFR; 42.5 mL/min/1.73 m^2^) or measured GFR (mGFR 42.8 mL/min/1.73 m^2^) were similar in CRIC and AASK, respectively, whereas individuals in ARIC were older (mean age 75.7 years) and had higher eGFR (mean 68.8 mL/min/1.73 m^2^) at baseline. The median urine protein/creatinine ratio (PCR) was 129.3 mg/g in CRIC and 80.0 mg/g in AASK, and the median urine albumin/creatinine ratio (ACR) was 10.7 mg/g in ARIC. AASK had 100% self-reported Black participants, whereas 41.7% of CRIC Study participants and 17.8% of ARIC study participants self-identified as Black individuals, respectively. The prevalence of diabetes was 49.1% in CRIC and 32.7% in ARIC, whereas no AASK study participants had diabetes. In CRIC, 675 individuals (38.2%) had CKD progression, as defined by incident end-stage renal disease (ESRD) or ≥ 50% decline in eGFR from baseline, over a mean follow-up of 6.8 years (Figure 1). In ARIC, 242 individuals (4.6%) had CKD progression by this definition over 6.4 years of mean follow-up. In AASK, 366 individuals (38.0%) had CKD progression, as defined by incident ESRD or doubling of serum creatinine concentration (equivalent to a ~57% decline in GFR), over a mean follow-up of 7.0 years.

### Metabolite profiling identifies markers of CKD progression in the CRIC Study.

**Figure 2:**
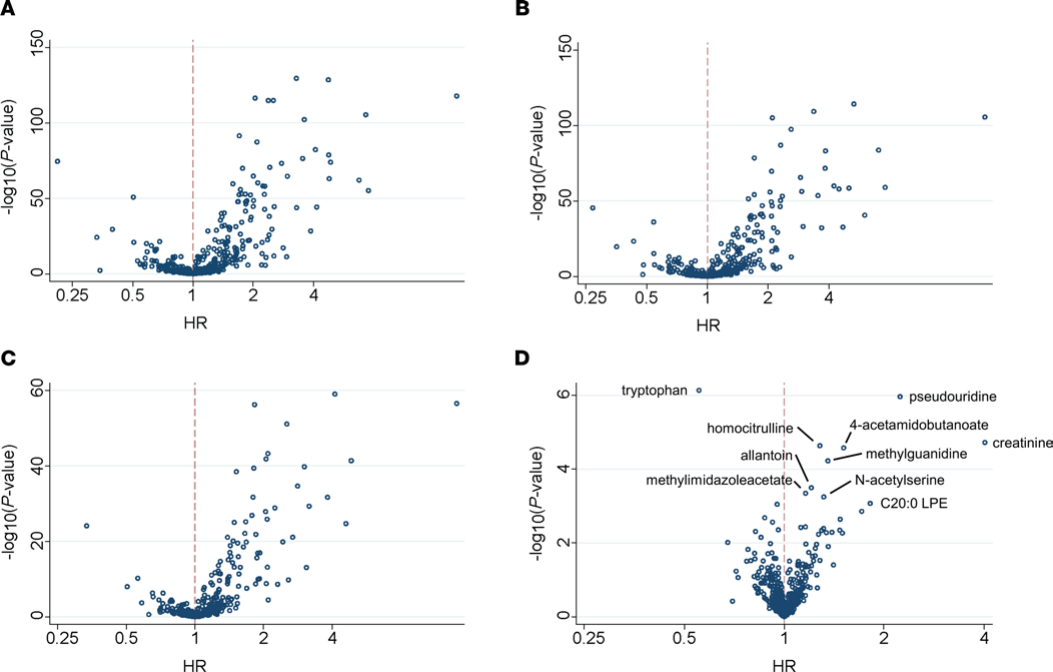
Metabolite profiling identifies markers of CKD progression in the CRIC Study. (**A**–**D**) Volcano plots depicting the HR (*x* axis) and *P* value (*y* axis) for CKD progression for each metabolite in Cox proportional hazards models adjusted for Model 1: age, sex, race, and study center (**A**); Model 2: Model 1 + BMI, systolic blood pressure, diabetes, cardiovascular disease, smoking, alcohol use, and *APOL1* genotype (**B**); Model 3: Model 2 + log PCR (**C**); and Model 4: Model 3 + eGFR (**D**). The 10 metabolites significantly associated with CKD progression at FDR < 0.05 in Model 4 are labeled in panel D (*n* = 1,773).

The volcano plot in Figure 2A shows the association of blood metabolites with CKD progression in Cox proportional hazards models adjusted for age, sex, race, and study center (Model 1) in CRIC. As shown, numerous metabolites were associated with CKD progression, with 267 metabolites significant at a FDR significance threshold of 0.05. Additional adjustment for comorbidities, specifically BMI, systolic blood pressure, diabetes, cardiovascular disease, smoking, alcohol use, and *APOL1* genotype had only a mild impact (Model 2, Figure 2B), and additional adjustment for proteinuria (Model 3, Figure 2C) had a moderate impact on the strength of associations. By contrast, additional adjustment for eGFR (Model 4, Figure 2D) markedly reduced the strength of metabolite associations with CKD progression, with the lowest *P* value of association at ~1 × 10^–7^, compared with ~ 1 × 10^–130^, ~ 1 × 10^–115^, and ~ 1 × 10^–60^ for Models 1, 2, and 3, respectively. Ten metabolites remained significantly associated with CKD progression at the chosen FDR threshold across all 4 models (Table 2 and Figure 2D). Except for tryptophan, all metabolites had a HR > 1, whereby higher metabolite levels were associated with an increased risk of CKD progression. Complete results for all metabolites are shown in Supplemental Table 1 (supplemental material available online with this article; https://doi.org/10.1172/jci.insight.161696DS1). In addition, stratified analyses by diabetes status for the 10 significant metabolites demonstrated similar results among individuals with or without diabetes (Supplemental Table 2).

### Replication of select metabolite markers of CKD progression in AASK and the ARIC study.

Of the 10 metabolites significantly associated with CKD progression in all models in CRIC, 9 were also measured in AASK and 8 were also measured in ARIC (Table 3). Three of these metabolites — pseudouridine, homocitrulline, and methylimidazoleacetate — were significantly associated with CKD progression in AASK and ARIC in fully adjusted Cox proportional hazards models similar to the fully adjusted model utilized in CRIC. Four metabolites — tryptophan, creatinine, 4-acetamidobutanoate, and N-acetylserine — had significant associations with CKD progression in either AASK or ARIC, but not both, in fully adjusted models. Methylguanidine was significantly associated with CKD progression in AASK but was not measured in ARIC. Allantoin did not replicate in either AASK or ARIC.

### Metabolite markers of CKD progression are highly correlated with eGFR.

**Figure 3:**
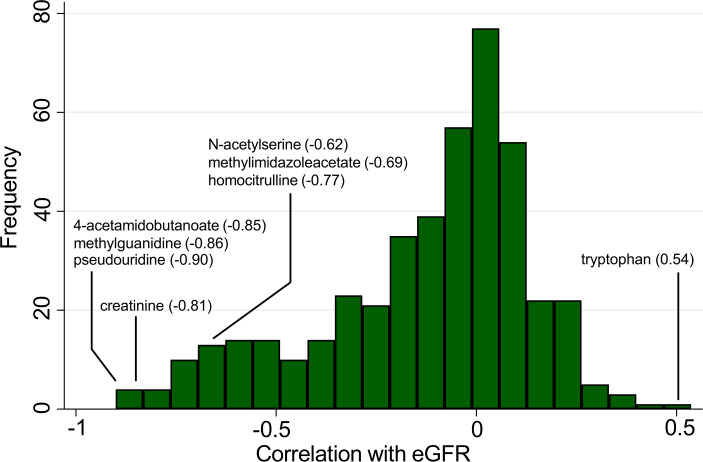
Metabolite predictors of CKD progression are correlated with eGFR in CRIC. Histogram showing frequency distribution of Spearman correlations of all measured metabolites with eGFR in CRIC (*n* = 1,773), with select metabolites associated with CKD progression labeled.

Kidney function affects the level of many circulating metabolites, in many cases because the metabolite undergoes renal clearance — i.e., through filtration, metabolism, secretion, or some combination thereof (10–12). As shown in Figure 3, blood metabolites demonstrated a left-skewed distribution toward stronger inverse correlations with eGFR in CRIC. Consistent with its direction of association with CKD progression, tryptophan had a positive correlation with eGFR (*r* = 0.54) and was the single most positively correlated metabolite with eGFR in CRIC. All of the other metabolites associated with CKD progression were inversely correlated with eGFR. The correlation coefficient for creatinine (measured by the metabolomics platform) with eGFR was –0.81. The inverse correlation with eGFR for several predictors of CKD progression in CRIC was even stronger, with *r* = –0.90 for pseudouridine, *r* = –0.86 for methylguanidine, and *r* = –0.85 for 4-acetamidobutanoate. Pseudouridine has already been highlighted as a candidate marker for glomerular filtration (13), and methylguanidine is a known breakdown product of creatinine. Homocitrulline (*r* = –0.77), methylimidazoleacetate (*r* = –0.69), and N-acetylserine (*r* = –0.62) were also strongly correlated with eGFR. In some cases, this may be indicative of other physiologic relationships with kidney function, where adjustment for eGFR has the potential to attenuate important biologic signals.

For example, homocitrulline is a nonstandard amino acid formed via the addition of cyanate to lysine’s free amino side chain, a process known as carbamylation (14). Because cyanate is a dissociation product of urea, protein carbamylation increases with progressive azotemia (elevated levels of urea and other nitrogenous compounds in blood). Indeed, the magnitude of the correlation between homocitrulline and blood urea nitrogen (BUN) concentration (*r* = 0.77) was as strong as the correlation between homocitrulline and eGFR (*r* = –0.77) and stronger than the correlation between homocitrulline and serum creatinine concentration (*r* = 0.68) in CRIC.

### Acy1 deficiency increases susceptibility to adenine-induced kidney injury.

N-acetylserine is an additional example where eGFR adjustment may attenuate a causal association. After undergoing glomerular filtration, N-acetylated amino acids are reabsorbed by the proximal tubule, where they undergo hydrolysis by aminoacylases, with return of free amino acids to the blood stream (15). In prior reports, children with mutations in *ACY1*, the gene encoding aminoacylase-1, were reported to have elevated urinary levels of several N-acetylated amino acids, including N-acetylserine (16–18). Kidney tissue is known to have the highest levels of *Acy1* expression, followed by the liver, with localization in the renal tubular epithelium (15). Based on immunofluorescence, we found that ACY1 is predominantly expressed in the proximal tubule (Supplemental Figure 1). To test whether N-acetylserine metabolism might play a functional role in CKD pathogenesis, we compared the impact of adenine diet in global *Acy1^–/–^* mice and *Acy1^+/+^* littermates. We found that absence of the ACY1 enzyme resulted in a trend for higher blood levels of its substrate, N-acetylserine, and that this difference was significantly magnified following 1 and 3 weeks on adenine diet (Supplemental Figure 2, A–C). *Acy1^–/–^* and *Acy1^+/+^* mice had similar levels of serum creatinine at baseline and 1 week, but ACY1 deficiency resulted in a greater elevation in serum creatinine on the adenine diet at 3 weeks (Supplemental Figure 2D). Consistent with this, *Acy1^–/–^* mice also had increased interstitial fibrosis relative to *Acy1^+/+^* mice on an adenine diet (Supplemental Figure 2, E and F).

## Discussion

The kidneys significantly impact circulating metabolites, particularly small, polar molecules that are normally excreted in urine. As kidney function worsens, blood levels of these metabolites increase. However, inverse correlation with eGFR does not necessarily indicate that a metabolite undergoes glomerular filtration, as the metabolites may undergo tubular secretion or metabolism within the kidney. Alternatively, alterations in diet, gut microbiota, or insulin sensitivity that accompany kidney disease may impact the metabolome (19). Finally, a metabolite could have an inverse relationship with eGFR if it is nephrotoxic or part of a causal pathway to eGFR decline. Rigorous adjustment for kidney function and its risk factors is appropriate for identifying the most robust metabolite biomarkers of CKD progression. However, these adjustments also have the potential to obscure or attenuate biologically informative associations.

Our fully adjusted analyses identified 3 metabolites — pseudouridine, methylimidazoleacetate, and homocitrulline — associated with CKD progression in CRIC, AASK, and ARIC. Pseudouridine, a modified nucleoside found in RNA, has been consistently highlighted in metabolomic studies of CKD and CKD progression in adults and children (10, 20, 21). It is strongly correlated with eGFR and, along with other proposed filtration markers, has been found to provide more accurate GFR estimation than creatinine or cystatin C alone (13). Subsequent measurement of its fractional excretion has shown that, in addition to undergoing glomerular filtration, pseudouridine undergoes partial net reabsorption (22). Notably, creatinine and its breakdown product methylguanidine were also associated with CKD progression, with replication for both in AASK (but not in ARIC), underscoring the ability of filtration markers to impart risk information even among individuals with directly measured GFR. Methylimidazoleacetate is the end product of histamine catabolism, with urinary levels utilized as an indicator of histamine turnover in the body (23). However, neither histamine nor methylimidazoleacetate has previously been implicated in CKD or its progression.

As noted, homocitrulline is the product of lysine carbamylation, a nonenzymatic process that increases with the rise in blood urea levels in CKD (14). Because this posttranslational modification alters protein structure, charge, and function, azotemia-induced protein carbamylation may be a deleterious causal factor in this context. Several studies have linked protein carbamylation with adverse outcomes — including erythropoietin resistance, heart failure, and mortality in ESRD (24–27) — and increased risk of CKD progression in a pilot study of 150 participants from the CRIC Study (28). These and other studies assessed protein carbamylation either by measuring carbamylated albumin levels or by assaying homocitrulline released after protein digestion (29, 30). Our results raise the possibility that free homocitrulline levels in blood may serve as a surrogate for protein carbamylation in CKD. More work is required to assess the relationship between free homocitrulline levels and other assays of protein carbamylation to determine which is the best predictor of disease progression and to determine if modulating azotemia and/or carbamylation can impact clinical outcomes.

Although the association between N-acetylserine and CKD progression observed in CRIC and ARIC was not significant in AASK, this signal has also been highlighted in other studies. For example, 3 N-acetylated amino acids, including N-acetylserine, were previously associated with progression to ESRD among 158 individuals with type 1 diabetes and CKD (20). Four N-acetylated amino acids, including N-acetylserine, were associated with ESRD or eGFR halving among 246 children with CKD stage 1 or 2 attributed primarily to glomerular disease or congenital abnormalities of the kidney or urinary tract (21). As outlined, N-acetylated amino acids undergo filtration and then proximal tubular absorption, where hydrolysis yields free (“salvaged”) amino acids that are returned to circulation (15). Thus, elevations in N-acetylserine or other acetylated amino acids in blood could reflect impaired proximal tubule metabolic function, providing additive information about kidney health beyond eGFR. More broadly, a growing body of literature supports the prognostic value of markers of kidney tubular health — for example, blood metabolite markers of kidney tubular secretory function (31, 32) or urinary protein markers of tubule cell injury (e.g., kidney injury molecule 1, monocyte chemoattractant protein 1) and dysfunction (e.g., α1-microglobulin, uromodulin/Tamm-Horsfall protein) (33).

In addition to providing insight on kidney tubular function, our exploratory studies raise the possibility that N-acetylserine, or its cognate enzyme ACY1, may be functional participants in CKD pathogenesis. We found that mice lacking ACY1 have a trend for elevated blood levels of N-acetylserine and that this biochemical impairment is markedly worsened with the onset of kidney injury. Furthermore, we found that mice lacking ACY1 had greater severity of adenine-induced injury relative to WT littermates, as evidenced by serum creatinine levels and tubulointerstitial histopathology. These findings raise several possibilities. First, N-acetylserine may be nephrotoxic, either in its circulating form or within the proximal tubule cell. Second, N-acetylserine may be a surrogate for other ACY1 substrates, including other N-acetylated amino acids, that are harmful in excess. A recent study showed that reducing the ratio of circulating N-acetylated amino acids to free amino acids by exogenous administration of ACY1 protein acutely improves glucose tolerance in mice (34). Third, given the diverse role of protein acetylation in cellular processes, including modulation of gene expression, protein stability, and signal transduction, impaired salvage of acetyl groups could be harmful to the proximal tubule (35). Fourth, impaired salvage of free amino acids could also be deleterious, although this seems less likely given the small quantitative contribution of acetylated amino acids to the total free amino acid pool. More work is required to disentangle these possibilities in model systems and to explore their potential relevance to human CKD. To date, only ~15 individuals (mostly children) with *ACY1* mutations have been reported, with a range of associated neurocognitive deficits but with no description of renal phenotypes (16–18, 36).

Prioritizing metabolites associated with CKD progression in fully adjusted models is one approach for biomarker and biologic discovery. Using an alternative approach, we previously conducted an analysis in AASK that started with the knowledge that genetic variants in *NAT8* are associated with CKD (37). We found that *NAT8* variants are associated with blood levels of 14 N-acetylated amino acids, which in turn were also associated with CKD progression, albeit at a relatively permissive statistical threshold (at *P* < 0.0036). N-acetylserine was not one of the *NAT8*-associated metabolites, raising the possibility that it is amino acid N-acetylation/deacetylation in general, rather than a single metabolite or enzyme, that is important in CKD pathogenesis. In a separate example of how genomics can enhance understanding of human metabolomic associations, Afshinnia et al. conducted a combined analysis of blood lipidomics and kidney tissue transcriptomics among individuals with type 2 diabetic kidney disease (38). This study showed that genes driving enhanced lipogenesis in the kidney could be responsible for the observed blood lipidomic alterations. Finally, rather than focus on longitudinal prediction of CKD progression, Chen et al. focused on metabolites that best discriminated cross-sectionally across different stages of CKD (39). This group identified 5-methoxytryptophan as a metabolite that is decreased with more severe CKD and then utilized mouse and cellular models to highlight a potential renoprotective role for this molecule. As these examples demonstrate, a range of transdisciplinary approaches can be utilized to augment the value of metabolomics studies of human CKD.

Our study has several limitations. We focused on only a single, baseline time point to maximize our power for biomarker discovery. We acknowledge that more effort is needed to track select metabolites of interest in the same individuals over time and, ideally, under different conditions to understand their dynamic response to diet, circadian oscillation, activity, and other factors (40–42). A thorough understanding of these details is a prerequisite for clinical utility. We focused on plasma because of our extensive experience with this biofluid, opportunities for replication with other blood-based data sets, and challenges in metabolite measurement across the wide range of urine dilution. Nevertheless, urine is an attractive biofluid for CKD risk assessment, and other groups have demonstrated its promise for metabolomic discovery (43–45). We have highlighted the increased confidence drawn from replication across 2 distinct LC-MS platforms, but we also acknowledge that neither platform — and, indeed, no available platform — provides comprehensive coverage of the metabolome. Finally, we examined the impact of global *Acy1* deletion in a commonly utilized CKD model. Because this initial analysis confirmed a specific role for ACY1 in N-acetylserine metabolism, as well as a potential role in renal protection, disease modeling following kidney-specific *Acy1* deletion is a logical future direction.

In summary, metabolite profiling in the CRIC, AASK, and ARIC studies identifies metabolites associated with CKD progression, including markers of glomerular filtration, azotemia, and proximal tubular metabolism. In addition to reinforcing a physiologic perspective on how the kidney interacts with the circulating metabolome, our study demonstrates the potential for metabolomics to motivate interest in select, potentially causal, metabolic pathways.

## Methods

### Study population.

Between 2003 and 2008, the CRIC Study recruited 3,939 individuals with mild to moderate kidney disease at 13 sites across the United States (6, 7). Study participants were between the ages of 21 and 74 years, with an eGFR of 20–70 mL/min/1.73 m^2^. By design, approximately 50% of the study subjects had diabetes, and individuals with polycystic kidney disease or on active immunosuppressive agents for glomerulonephritis were excluded. A total of 1,800 randomly selected participants who attended the year 1 visit underwent blood metabolomic profiling using the Broad Institute Metabolomics Platform (12). The AASK was a prospective cohort of 1,094 African Americans with kidney disease (8). Originally a trial of blood pressure management, participants were randomized to a higher versus lower blood pressure target, and an ACE-inhibitor, a calcium channel blocker, or a beta blocker; the trial phase was followed by a cohort phase, with the full follow-up encompassing an average of 16 visits over up to 12 years (46, 47). A total of 962 individuals at the baseline visit underwent blood metabolomic profiling by Metabolon Inc. The ARIC study is composed of 15,792 individuals between the ages of 45 and 64 years prospectively enrolled from 4 communities in the United States (9). Visit 1 was conducted between 1987 and 1989, and follow-up is ongoing. For this study, we included 5,305 participants without ESRD at visit 5 (between 2011 and 2013) who had blood metabolomic profiling by Metabolon Inc.

### Metabolomics.

Detailed methods, including characterization of technical and intraperson analyte variation among individuals with CKD, for both the Broad Institute and Metabolon metabolomics methods have been published (12).

For CRIC, fasting samples stored at –80°C underwent profiling at the Broad Institute using a combination of 3 LC-MS injections. Data were acquired using LC-MS systems composed of Nexera X2 U-HPLC systems (Shimadzu Scientific Instruments) coupled to Q Exactive/Exactive Plus orbitrap mass spectrometers (Thermo Fisher Scientific). Positively charged polar analytes were measured in samples extracted with acetonitrile/methanol/formic acid and separated by hydrophilic interaction LC (HILIC), with MS analyses carried out using positive ion mode electrospray ionization (ESI); this method was also used to measure N-acetylated amino acids in 10 μL of mouse plasma samples. Negatively charged polar analytes were measured in samples extracted with methanol and separated on an NH2 column, with MS analyses in negative ion mode ESI. Positively charged lipids were measured in samples extracted with isopropanol and separated on a C8 column, with MS analyses carried out using positive ion mode ESI. Raw metabolomics data were processed using TraceFinder (Thermo Fisher Scientific), and identification of metabolite peaks was conducted by matching measured retention times and masses to mixtures of reference metabolites analyzed in each batch and to an internal database of compounds that have been characterized.

For AASK and ARIC, fasting samples stored at –80°C underwent profiling at Metabolon. Data were acquired using LC-MS systems composed of ACQUITY UPLC systems (Waters) coupled to Q Exactive orbitrap mass spectrometers (Thermo Fisher Scientific). The Metabolon process spikes samples with recovery standards and removes proteins with methanol. The extract is then divided into 5 fractions: 2 for separate reverse phase ultraperformance LC-MS/MS (RP/UPLC-MS/MS) with a positive ion mode ESI, 1 for RP/UPLC-MS/MS with negative ion mode ESI, 1 for HILIC-UPLC-MS/MS with negative ion mode ESI, and 1 sample for back-up. Experimental peaks are identified by retention time/index, mass/charge ratio, and chromatographic data; to be labeled, compounds must match on all 3 criteria with the purified, authenticated standards in the Metabolon library.

### Metabolomic data processing.

Drug metabolites and metabolites missing in > 50% of samples were excluded from the present analysis, leaving 443 metabolites for evaluation in CRIC (median percentage missing, 0%; range 0%–47%). Missing values were imputed with the lowest observed level, as has been done previously (48, 49). Metabolites were log_2_-transformed to normalize their skewed distributions. Only the 10 metabolites significant in CRIC were evaluated in AASK and ARIC.

### Outcomes.

The primary study outcome was CKD progression. In CRIC and ARIC, this was defined as incident ESRD and/or decline in eGFR by ≥ 50%. In AASK, this was defined as incident ESRD and/or doubling of serum creatinine concentration (equivalent to a ~57% decline in GFR). In CRIC, eGFR was calculated from serum creatinine and serum cystatin C concentrations using the CRIC Study equation (50). In ARIC, eGFR was calculated from serum creatinine and serum cystatin C concentrations using the Chronic Kidney Disease Epidemiology (CKD-EPI) Collaboration equation (51). In CRIC and AASK, ESRD was ascertained per protocol throughout the study periods. In ARIC, ESRD was ascertained through linkage to the United States Renal Data System (USRDS) in December 2018 (52).

### Covariates.

For each cohort, covariates were assessed at the same visit as metabolomic profiling. In AASK, GFR was measured by the urinary clearance of ^125^I Iothalamate. PCR was determined from 24-hour urine collection or random spot measures in CRIC and from random spot measures in AASK. ACR was determined from random spot measures in ARIC. In CRIC, diabetes was defined as self-reported use of insulin or oral hypoglycemic medications, fasting blood glucose ≥ 126 mg/dL or a nonfasting level ≥ 200 mg/dL, or an HbA1c ≥ 6.5%. AASK did not enroll any study participants with diabetes. In ARIC, diabetes was defined as the current use of a diabetes medication, fasting blood glucose ≥ 126 mg/dL or a nonfasting level ≥ 200 mg/dL, or self-report of a physician’s diagnosis of diabetes. History of cardiovascular disease was self-reported in CRIC and AASK. In ARIC, prevalent cardiovascular disease at visit 5 was a combination of self-reported disease at visit 1 and adjudicated events between visit 1 and visit 5.

### Acy1^–/–^ mice.

*Acy1^+/–^* mice were obtained from MMRRC at UC Davis (MMRRC_046467-UCD). These mice were generated in a C57BL/6N background using CRISPR (https://www.mmrrc.org/catalog/sds.php?mmrrc_id=46467). In our laboratory, *Acy1^+/–^* mice were bred to generate *Acy1^–/–^* mice and *Acy1^+/+^* littermates. Because Acy1 is primarily expressed in the proximal tubular epithelium, we used an adenine diet–induced CKD model, which is characterized by tubulointerstitial injury. *Acy1^–/–^* or *Acy1^+/+^* male mice (8–10 weeks old) were placed on normal or 0.2% adenine diet (TD.130900, Envigo) for 3 weeks before sacrifice. Serum and kidneys were collected for further analysis.

### Mouse phenotyping.

Serum creatinine was measured using LC-MS. In brief, 10 μL of mouse plasma was extracted with 90 μL acetonitrile/methanol (3:1 ratio) containing creatinine-d3 (Sigma-Aldrich). After centrifugation (11,180*g* for 10 minutes at room temperature), supernatant was separated using a 2.1 × 150 mm Atlantis HILIC Silica 3 μm column (Waters). The peaks for creatine (transition 114.15/44.04) and creatinine-d3 (transition 117.15/47.04) were monitored in the positive ion mode on a TSQ Quantiva Triple Quadrupole MS (Thermo Fisher Scientific). For immunofluorescence, mouse kidney samples were fixed with 4% paraformaldehyde overnight, embedded in paraffin, and sectioned onto slides. Primary antibodies were used as follows: rabbit anti-ACY1 (PA5-81310, lot no. UI2841839, Invitrogen); mouse anti-AQP1 (sc25287, lot no. F1019, Santa Cruz Biotechnology Inc.); mouse anti-AQP2 (sc515770, lot no. I0519, Santa Cruz Biotechnology Inc.); and mouse anti-THP (sc271022, lot no. B1219, Santa Cruz Biotechnology Inc.) (all diluted 1:500, incubated overnight). After washing the tissue, slides were incubated for 1 hour at room temperature in the dark with the secondary antibody (donkey anti–rabbit IgG–conjugated Alexa Fluor 488 and donkey anti–mouse IgG–conjugated Alexa Fluor 568, both diluted 1:200). For IHC, mouse kidney samples were sectioned onto slides and incubated overnight with primary rabbit anti-ACY1 (PA5-81310, lot no. UI2841839, Invitrogen, diluted 1:500). After washing the tissue, slides were incubated for 1 hour at room temperature with secondary goat anti-rabbit HRP (65-6120, Thermo Fisher Scientific). DAB kit (34065, Thermo Fisher Scientific) was used according to the manufacturer’s instruction. For assessment of kidney fibrosis, Sirius red staining to detect collagen fibers was performed according to the manufacturer’s protocol (ab150681, Abcam). Briefly, tissue sections were deparaffinized and hydrated in distilled water. Adequate Picrosirius red solution was applied to completely cover the tissue section and incubated for 60 minutes. Afterward, sections were rinsed quickly in 2 changes of acetic acid solution and then absolute alcohol. Finally, slides were cleared and mounted in synthetic resin. The area of interstitial fibrosis (stained with Sirius red) was identified, after excluding the vessel area from the region of interest, as the ratio of interstitial fibrosis/total tissue area and expressed as the percentage of fibrotic area using ImageJ software (NIH). For immunoblotting, mouse kidney samples were homogenized using RIPA buffer with protease inhibitors (78429, Thermo Fisher Scientific). Protein extracts were separated by electrophoresis on SDS-PAGE and transferred to nitrocellulose membranes, which were then washed and incubated with blocking buffer (5% nonfat milk in TBS) for 1 hour at room temperature. The membranes were then incubated overnight at 4°C with the primary antibodies: mouse anti-fibronectin antibody (F7387, lot no. 0000137718, Sigma-Aldrich), mouse anti–α-SMA antibody (sc32251, lot no. C0521, Santa Cruz Biotechnology Inc.), and mouse anti–β-actin antibody (sc47778, lot no. J1119, Santa Cruz Biotechnology Inc.) (all diluted 1:500, incubated overnight). The next day, the membranes were washed and incubated with the appropriate HRP-coupled secondary antibodies, and signals were detected with ECL (Pierce, Thermo Fisher Scientific). Expression of proteins was quantified by densitometry using ImageJ software (NIH).

### Statistics.

Baseline characteristics were summarized using mean ± SD or proportions, with baseline defined as a year-1 visit in CRIC, randomization in AASK, and visit 5 in ARIC. The relationship between metabolites and eGFR was assessed using Spearman correlations. Cox proportional hazards models were used to estimate the association between metabolites and CKD progression in a series of sequentially adjusted models: Model 1 adjusted for age, sex, race, and study center; Model 2 adjusted for variables in Model 1 plus BMI, systolic blood pressure, diabetes, cardiovascular disease, smoking, alcohol use, and *APOL1* genotype; Model 3 adjusted for variables in Model 2 plus log-transformed PCR; Model 4 adjusted for variables in Model 3 plus eGFR. Statistical significance was based on FDR < 0.05 (53). In AASK and ARIC, only Model 4 was run. mGFR was substituted for eGFR in AASK, and there was no adjustment for race, diabetes, alcohol use, or *APOL1* genotype (because AASK only enrolled Black individuals, no study participants had diabetes, genotyping was only conducted in a subset of study participants, and information on alcohol use was missing); in ARIC, there was no adjustment for alcohol use, and log-transformed ACR was substituted for log-transformed PCR. In sensitivity analyses, analyses were repeated stratified by the presence of diabetes, and an interaction term was evaluated by including the product term of diabetes and metabolite in the full model. For mouse studies, statistical significance in 2-tailed Student’s *t* tests was defined as *P* < 0.05. All analyses were run using STATA SE 17.0.

### Study approval.

All participants provided written informed consent, and the study adhered to the Declaration of Helsinki and was approved by the IRBs of the Perelman School of Medicine at the University of Pennsylvania and Johns Hopkins University. All animal studies were approved by the IACUC of the Massachusetts General Hospital and conducted under their guidelines.

## Author contributions

DW, SK, CYH, SSW, CMR, MRD, PLK, RSV, JC, HIF, MEG, and EPR conceptualized the studies. DW and WZ conducted in vivo experiments. JAP and CBC acquired the metabolomics data. BY, JH, CYH, AP, PR, JS, RT, JC, and MEG acquired the clinical data and provided resources. ZZ, AS, LZ, DX, HS, and CBC analyzed the data. EPR wrote the manuscript with input from all authors.

## Supplementary Material

Supplemental data

ICMJE disclosure forms

Supplemental table 1

## Figures and Tables

**Table 1:**
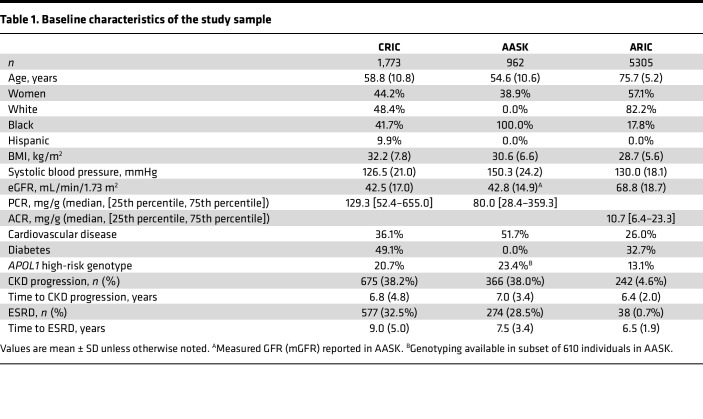
Baseline characteristics of the study sample

**Table 2:**
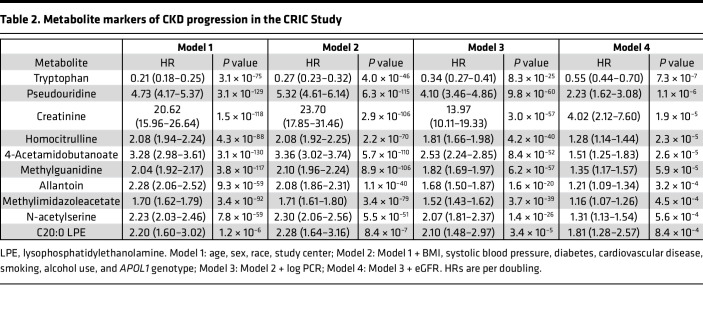
Metabolite markers of CKD progression in the CRIC Study

**Table 3:**
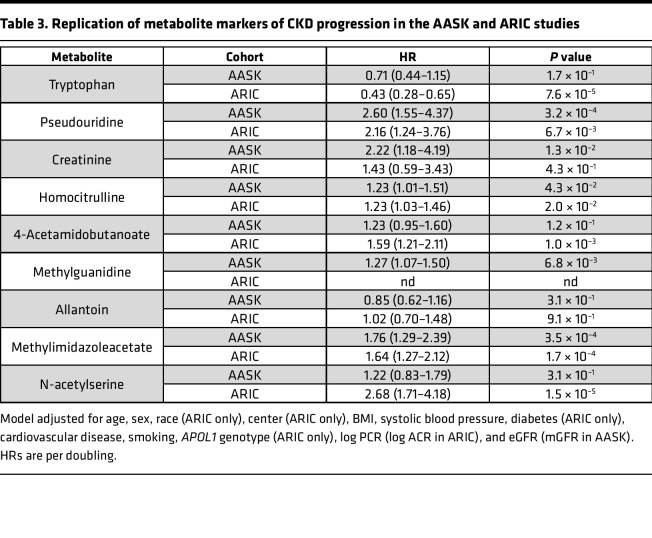
Replication of metabolite markers of CKD progression in the AASK and ARIC studies

## Version 1 09/01/2022

In-Press Preview

## Version 2 10/24/2022

Electronic publication
