# Supplementary material for: Metabolite profiling of CKD progression in the chronic renal insufficiency cohort study
Source: JCI Insight. 2022 Oct 24;7(20):e161696. doi: 10.1172/jci.insight.161696 (PMC9714776; doi:10.1172/jci.insight.161696)
Supplement: Supplemental data [file jciinsight-7-161696-s072.pdf]

Supplemental material

Supplemental Figure legends

Supplemental Figure 1. ACY1 is expressed in the proximal tubule with colocalization with aquaporin 1, but not Tamm-Horsfall protein or aquaporin 2. Representative photographs of immunofluorescence for (**A**) ACY1 (left), AQP1 (middle), and merge (right), (**B**) ACY1 (left), Tamm-Horsfall protein (middle), and merge (right), and (**C**) ACY1 (left), AQP2 (middle), and merge (right) of kidney cortex from C57Bl/6J mice (scale bar: 100 µm). Shown are representative images from 1 of 2 mice.

Supplemental Figure 2. *Acy1-/-* mice have increased susceptibility to adenine induced kidney injury. (**A**) Representative photographs of immunofluorescence for ACY1 (left), AQP1 (middle), and merge (right) of kidney cortex from *Acy1+/+* and *Acy1-/-* mice (scale bar: 100 µm). Shown are representative images from 1 of 2 mice for each genotype. (**B**) Representative photomicrographs of immunohistochemistry for ACY1 of kidney cortex from *Acy1+/+* and *Acy1-/-* mice (scale bar: 100 µm). Shown are representative images from 1 of 2 mice for each genotype. (**C**) Blood N-acetylserine and (**D**) creatinine levels in *Acy1+/+* and *Acy1-/-* mice at baseline and after 1 and 3 weeks of adenine or control diet (*n*=3-5 per group). (**E**) Sirius red staining of kidney cortex from *Acy1+/+* and *Acy1-/-* mice after 3 weeks of adenine or control diet (scale bar: 100 µm); graph shows results quantitated for *n*=5 per group. (**F**) immunoblots of kidney cortex for fibronectin, α-smooth muscle actin, and β-actin from *Acy1+/+* and *Acy1-/-* mice after 3 weeks of adenine diet; graphs show results quantitated for *n*=4 per group. Data represent the mean ± SEM. **P*-values from 2-sided Student’s t-test (C-F).

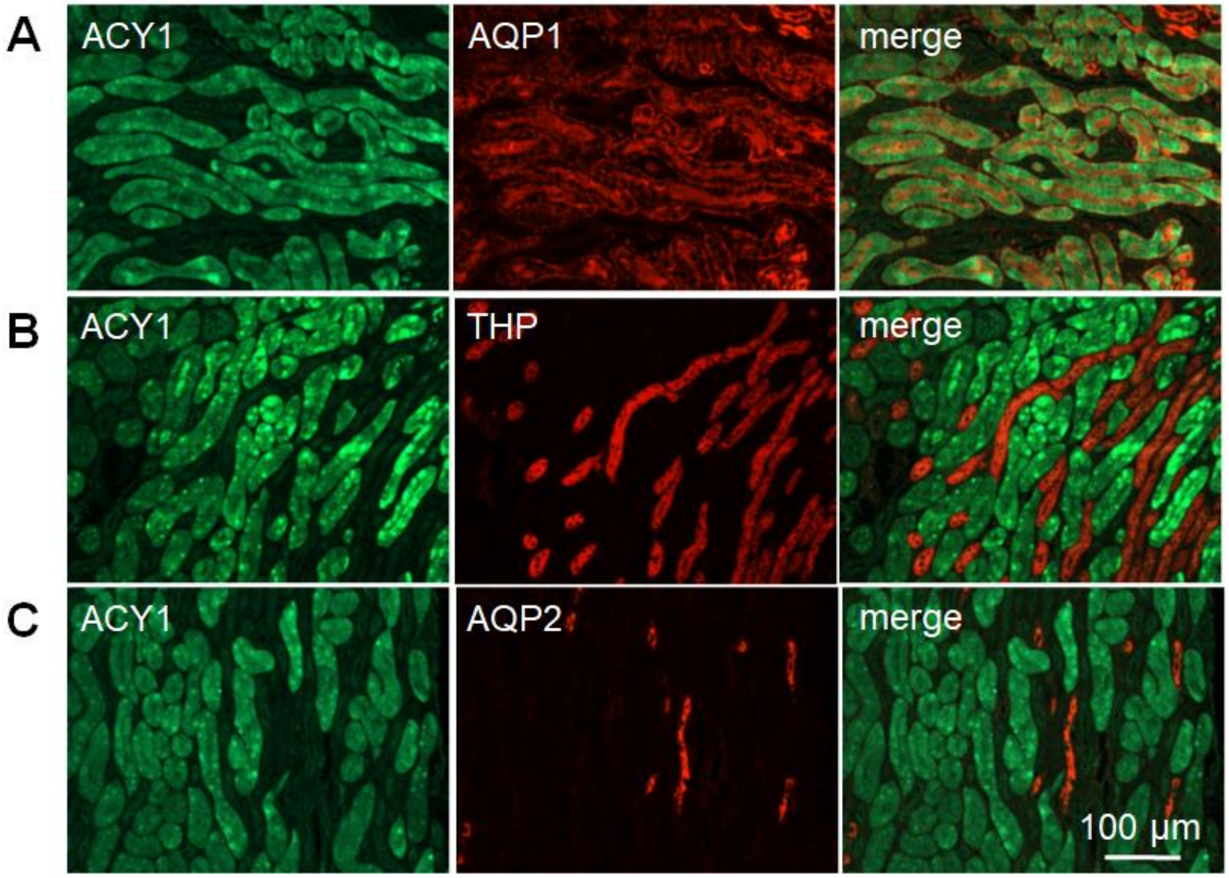

Supplemental Figure 1

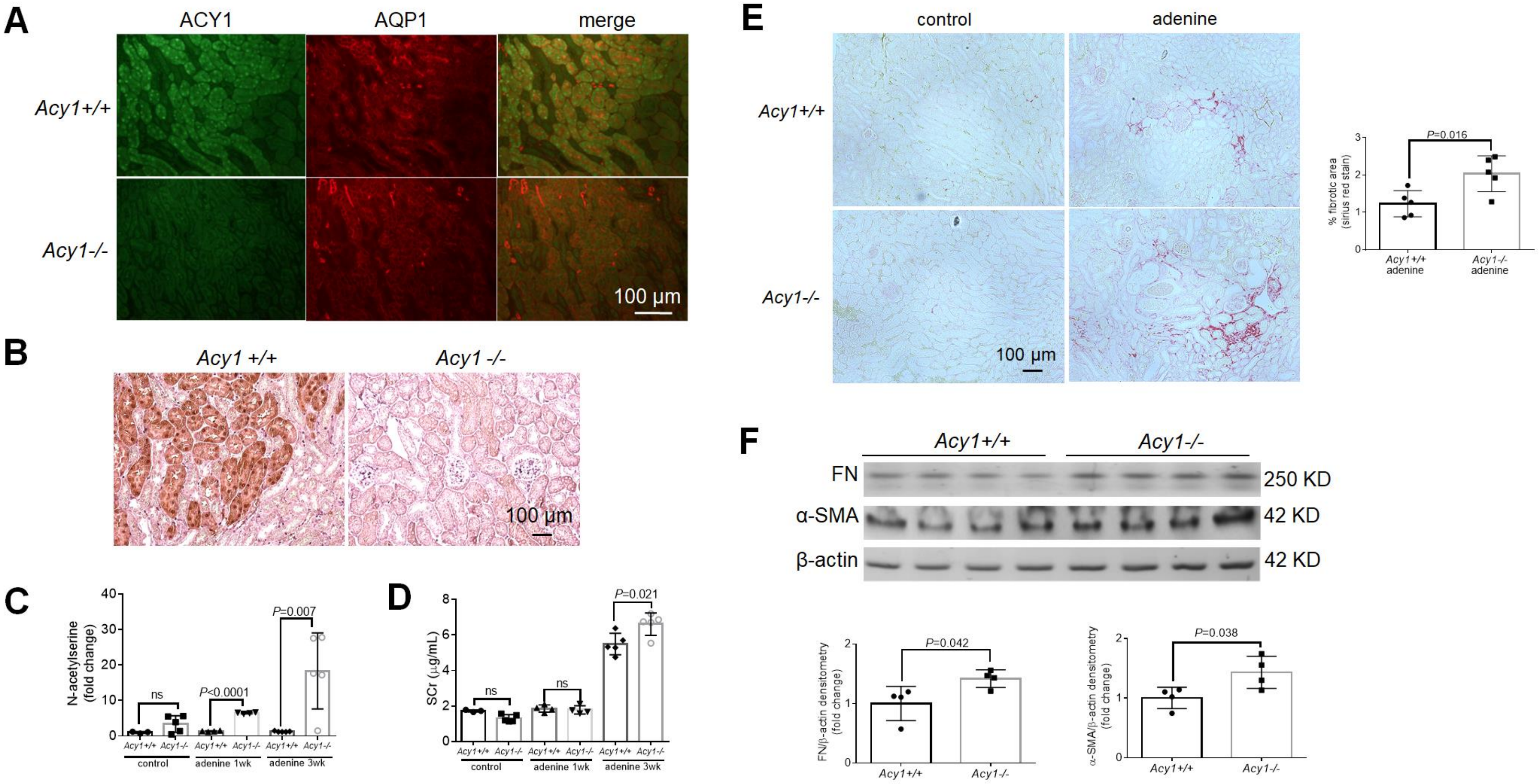

Supplemental Figure 2

Supplemental Table 1. All metabolite associations with CKD progression in the CRIC study

See attachment

Supplemental Table 2. Metabolite markers of CKD progression in the CRIC study stratified by diabetes status

	No Diabetes		Diabetes		
**Metabolite**	**HR**	***P*-value**	**HR**	***P*-value**	**Interaction *P*-value**
tryptophan	0.55 (0.38 - 0.79)	1.23E-03	0.56 (0.43 - 0.73)	1.44E-05	9.63E-01
pseudouridine	2.17 (1.48 - 3.19)	8.14E-05	2.26 (1.61 - 3.18)	2.26E-06	7.92E-01
creatinine	6.14 (2.91 - 12.99)	2.02E-06	3.35 (1.73 - 6.51)	3.49E-04	3.65E-02
homocitrulline	1.30 (1.12 - 1.52)	7.04E-04	1.27 (1.11 - 1.44)	4.02E-04	7.16E-01
4-acetamidobutanoate	1.42 (1.12 - 1.81)	4.42E-03	1.56 (1.27 - 1.91)	2.40E-05	4.19E-01
methylguanidine	1.40 (1.18 - 1.65)	8.91E-05	1.32 (1.13 - 1.55)	5.34E-04	4.28E-01
allantoin	1.13 (0.96 - 1.32)	1.37E-01	1.25 (1.10 - 1.41)	4.06E-04	2.94E-01
methylimidazoleacetate	1.19 (1.07 - 1.33)	1.32E-03	1.14 (1.03 - 1.25)	7.47E-03	4.03E-01
N-acetylserine	1.29 (1.03 - 1.62)	2.80E-02	1.33 (1.11 - 1.59)	2.27E-03	8.28E-01
C20:0 LPE	2.84 (1.58 - 5.10)	4.70E-04	1.47 (0.96 - 2.24)	7.65E-02	6.44E-02

Models adjusted for age, sex, race, study center, BMI, systolic blood pressure, cardiovascular disease, smoking, alcohol use, *APOL1* genotype, log PCR, and eGFR. HRs are per doubling.

Supplemental Acknowledgments

The CRIC Investigators are Lawrence J. Appel, Alan S. Go, Jiang He, Robert G. Nelson, Panduranga S. Rao, Mahboob Rahman, Vallabh O. Shah, Raymond R. Townsend, and Mark L. Unruh. The CKD Biomarker consortium members include Dr. Vasan S. Ramachandran, Dr. Jeffrey Schelling, Dr. Michelle Denburg, Dr. Susan Furth, Dr. Bradley Warady, Dr. Joseph Bonventre, Dr. Sushrut Waikar, Dr. Venkata Sabbisetti, Dr. Josef Coresh, Dr. Morgan Grams, Dr. Casey Rebholz, Dr. Alison Abraham, Dr. Chirag Parikh, Dr. Steven Coca, Dr. Eugene Rhee, Dr. Paul L. Kimmel, Dr. Michael G. Shlipak, Dr. Mark Sarnak, Dr. Orlando M. Gutiérrez, Dr. Joachim Ix, Dr. Ruth Dubin, Dr. Tom Hostetter, Dr. Rajat Deo, Dr. Harold I. Feldman, Dr. Sarah J Schrauben, Dr. Dawei Xie, Dr. Haochang Shou, Mr. Shawn Ballard, Ms. Krista Whitehead, Dr. Heather Collins, Dr. Jason H. Greenberg, and Dr. Peter Ganz.
